# Safeguarding sustenance: Singapore’s strategic commitment to enhancing food security through advancing food research and innovation

**DOI:** 10.1098/rstb.2024.0164

**Published:** 2025-09-18

**Authors:** Aaron Zongwei Li, Ying Tong Yeo, Wei Ning Chen

**Affiliations:** ^1^ School of Chemistry, Chemical Engineering and Biotechnology, Nanyang Technological University, Singapore, Singapore

**Keywords:** food security, technology innovation, alternative foods

## Abstract

The article examines Singapore’s proactive approach to addressing its food security challenges amid limited natural resources and external pressures such as climate change and global market volatility. In light of the ‘30 by 30’ goal, which aspires to produce 30% of the nation’s nutritional needs in Singapore by 2030, the article outlines Singapore’s drive for innovation through a comprehensive framework based on technological innovation, regulatory agility and collaborative partnerships that underpin Singapore’s food security strategy. Key factors for success include strategic leadership, robust investments in research and development, the nurturing of a skilled workforce and a focus on sustainable practices and market acceptance. Lessons and parallels with global leaders in different sectors of food innovation are also explored. By positioning itself as a hub for cutting-edge food technologies and sustainable practices, Singapore aims to set a benchmark for resilience in food systems that can serve as a model for other nations confronting similar issues.

This article is part of the theme issue ‘Transforming terrestrial food systems for human and planetary health’.

## 1. Introduction

Singapore, a compact and highly developed city-state with a small geographical footprint of approximately 735 km^2^ and a population of 6.04 million as of June 2023 [1], is one of the most densely populated countries in the world [2]. With the population expected to grow, so too will the demand for food [3,4]. Having scarce arable land (<1%) [5] and limited natural resources, Singapore can only rely on importing food to sustain this demand. However, global phenomena such as climate change [6], volatile international trade networks [7], repercussions of pandemics—whether of human [8], animal (affecting livestock) [9] or plant (affecting agricultural crops) [10] nature—complicate this endeavour. Hence, securing enough food for Singapore becomes an issue of paramount importance to tackle.

Food security, as agreed upon by the World Food Security in 2012, is defined as ‘when all people at all times have access to sufficient, safe and nutritious food that meets the dietary needs and food preferences for a productive and healthy life’ [11, p. 623].

According to the Singapore Food Statistics Report from 2021, Singapore’s annual per capita consumption of food items includes approximately 390 eggs, 100 kg of vegetables, 22 kg of seafood, 62 kg of meat (including chicken, pork, beef and mutton) and 76 kg of fruits [12]. This amounts to a national demand of about 2356 million eggs, 604 000 tonnes of vegetables, 132 880 tonnes of seafood, 374 480 tonnes of meat and 459 040 tonnes of fruits per year. Within the 2023 report, the total amount of eggs, vegetables, seafood, meat and fruits supplied was 2161 million eggs, 547 400, 126 500, 380 300 and 404 800 tonnes, respectively.

Of this amount, Singapore presently imports more than 90% of it. Although the government has actively diversified its food import sources to over 180 countries [13], the nation remains heavily reliant on a few key countries. Notably, Malaysia is a significant supplier of hen shelled eggs (>50%) and fruits and vegetables (>33%), while Brazil is a major source of meat (>50%). This dependence on a few key countries exposes Singapore to potential risks associated with global disruptions, such as pandemics or geopolitical conflicts.

In response to these difficult challenges, Singapore has initiated a strategic and scientifically rigorous campaign to bolster its food security through the integration of advanced food research and innovation. Central to this mission is the ‘30 by 30’ initiative [14], a visionary goal by the Singapore Government in 2019 to produce 30% of the nation’s nutritional needs by 2030, underscoring Singapore’s dedication to enhancing self-sufficiency and resilience within its food systems.

As Singapore navigates the complexities of securing its food supply, it has become evident that a multifaceted approach is essential to achieving sustainable success. The nation’s strategy revolves around several critical pillars that together form the foundation of its innovative food security framework. Singapore’s strategic framework is predicated on three core pillars: ample resources for research, collaborative partnerships and agile regulatory frameworks. These pillars are not isolated; they are deeply interconnected, each reinforcing the other to create a resilient and adaptive food ecosystem.

Through this article, we aim to provide an overview and analysis of Singapore’s strategic initiatives in enhancing food security through scientific research and technological innovation. By evaluating the primary drivers behind these initiatives, the cutting-edge technological advancements being pursued and the synergistic collaborative frameworks that underpin this ambitious agenda, we hope to elucidate how Singapore is positioning itself at the forefront of global food security efforts, offering a model of resilience and sustainability for other nations facing analogous challenges.

## 2. Pillars for innovation

**Figure 1.**
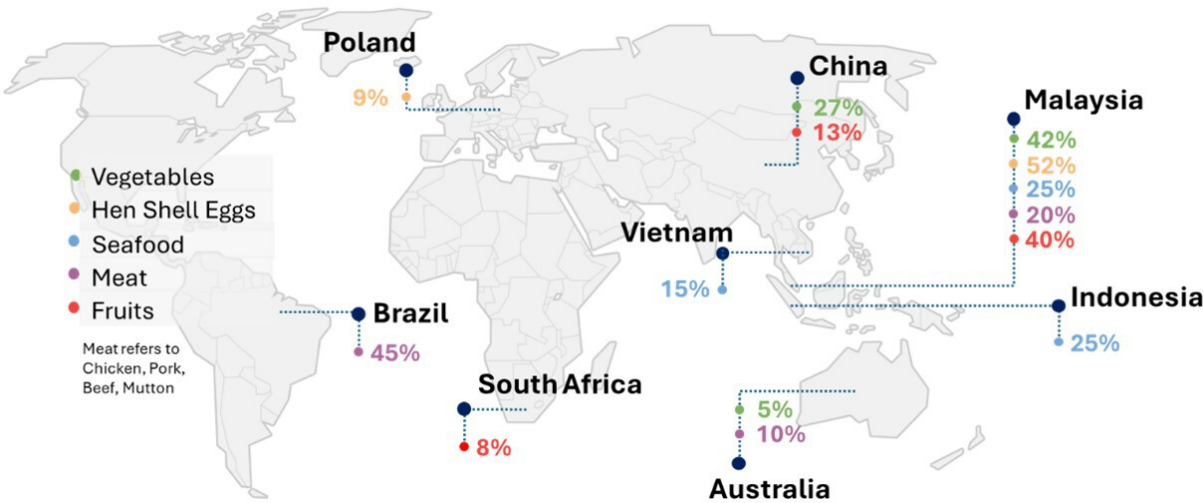
Singapore food supply sources. Adapted from Singapore Food Statistics Report 2021 [12].

Singapore’s approach to food security addresses immediate challenges while laying the groundwork for long-term sustainability and innovation. Upon closer assessment, we identify three key areas that encapsulate the essential factors driving Singapore’s success in advancing food research and innovation (figure 1).

### (a) Ample research and development resources

**Figure 2.**
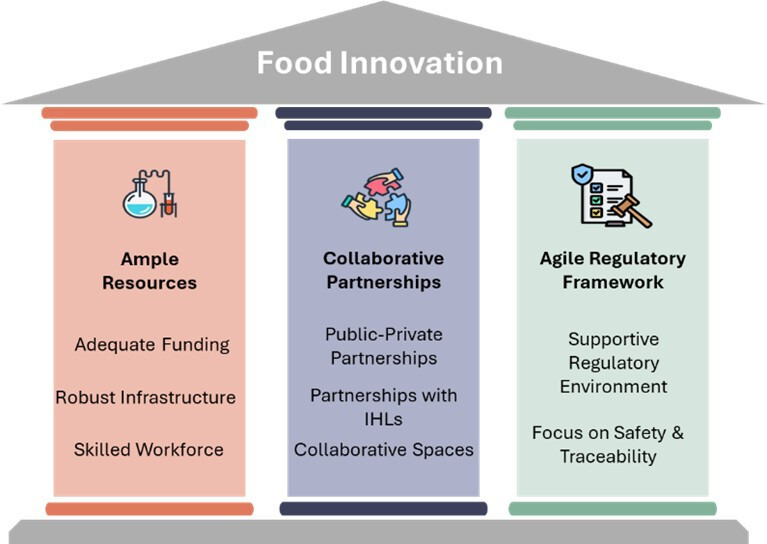
Singapore’s strategic framework comprising three core pillars for advancing food research and innovation.

In research investigating the critical factors for success in research and development (R&D) projects, Pinto *et al.* identified adequate funding, robust infrastructure and skilled manpower as top factors for success [15]. Singapore takes a similar approach through the provision of research funding, establishment of infrastructure and advancement of its workforce. These strategic efforts underscore the nation’s commitment to advancing food security. Continuous investment in R&D is the backbone of innovation (figure 2; [16,17]).

#### (i) Research funding

On the front of research funding, the Singapore Food Story R&D programme was jointly developed in 2020 by the Singapore Food Agency (SFA), the leading food authority in Singapore and Agency for Science, Technology and Research (A*STAR), a statutory board under the Ministry of Trade and Industry (MTI) that focuses on advancing the economy through science and technology innovations [18]. This programme—with SGD (Singapore dollars) 309 million in funding—aims at bolstering our food security and resilience through quality research and innovation across three broad themes, namely aquaculture, sustainable urban agriculture, future foods, food safety, with funding allocated at different stages of research [19].

Seed grants target nascent research with a technology readiness level of 1–3, focusing on knowledge creation and catalysing early-stage innovation. These grants typically fund up to SGD 1 million and last for up to 18 months [20]. Additionally, larger grants offer a higher quantum of up to SGD 8 million, and these are usually well-aligned with the public sector and have collaborations with industry partners [20]. These grants support more established research, focusing on prototyping and improving scale-up and production to accelerate new food product development and market entry.

#### (ii) Robust research infrastructure

Another critical aspect of Singapore’s strategy to bolster food security through innovation is its investment in state-of-the-art research infrastructure [21,22]. This provides the essential tools, facilities and resources needed to support advanced research and accelerate the development of new food technologies. Some of the key components of Singapore’s R&D infrastructure include the following.

#### Specialized research facilities

Singapore has developed several specialized research facilities dedicated to advancing food technology and innovation. This helps to not only provide niche areas of expertise but also seeks to consolidate resources in more targeted research areas. Some of these research facilities include:

— *Food Innovation & Resource Centre (FIRC)*: located at Singapore Polytechnic, the FIRC offers comprehensive facilities for food research, including pilot plant facilities, sensory evaluation laboratories and analytical testing services. These resources support food companies in developing and scaling new products, from initial concept through to market-ready solutions [23];
— *Singapore Institute of Food and Biotechnology Innovation (SIFBI)*: SIFBI serves as a hub for cutting-edge research in food science and biotechnology. Equipped with advanced laboratories and technology platforms, SIFBI focuses on areas such as alternative proteins, food safety and sustainable food production. Its facilities are designed to support both fundamental research and applied science, facilitating the translation of discoveries into commercial applications [24]; and
— *Aquaculture Innovation Centre (AIC)*: housed at Temasek Polytechnic, the AIC is dedicated to advancing aquaculture technologies, supporting research in sustainable fish farming practices, including advanced breeding techniques, disease management and efficient feed systems. The AIC aims to boost local seafood production and reduce dependence on imports, contributing significantly to Singapore’s food security goals [25,26].

#### Innovation hubs and incubators

Innovation hubs and incubators play a crucial role in fostering the growth of start-ups and Small and Medium Entreprises (SMEs) within the food sector (table 1). They provide essential resources, mentorship and support to help these entities develop and scale innovative food technologies.

**Table 1.** Examples of innovation hubs and incubators that support food start-ups in Singapore.

accelerator/incubator/ innovation hub	focus area	key offerings	support provided	impact
Innovate360	helping food start-ups scale and speed up go-to-market	specialized laboratories, pilot testing facilities, commercial kitchens	business development support, market research, branding, regulatory guidance	accelerates commercialization of new food products and technologies
Grow	first agri-foodtech start-up accelerator in southeast Asia	collaborative environment, laboratory space, technical support	mentorship programmes, funding opportunities, industry connections	backed by AgFunder and can help SMEs expand internationally
HatchBlue	aquaculture and seafood industry	state-of-the-art R&D facilities for sustainable aquaculture	mentorship, industry expertise, funding opportunities	advances practices for efficient and sustainable seafood production, enhancing food security
The Hatchery	agri-food technology, industrial and synthetic biology, human and veterinary sciences	laboratory facilities, technical support	business mentoring, regulatory guidance, market positioning, scaling strategies	developed and supported by Temasek Life Science Accelerator supports start-ups through the entire process, from idea-to-market in food technology
SAIL	agri-food ecosystem, from farming to agri inputs to supply chain digitalization to downstream R&D	matching solution providers with problem statements	consultancy, monitoring, facilitating	funded by Enterprise Singapore and managed by Nanyang Technological University

These innovation hubs, accelerators and incubators not only provide critical resources and support but also foster a collaborative environment that encourages knowledge exchange and partnership. By nurturing start-ups and facilitating industry connections, they play a significant role in driving food innovation and enhancing Singapore’s food security [27,28].

Investment in R&D is a cornerstone of Singapore’s strategy to enhance food security through innovation. Recognizing the critical role that science and technology play in transforming the food landscape, Singapore has made significant financial and institutional commitments to foster a robust R&D ecosystem. This investment is not just about creating new technologies, but more importantly, it builds the infrastructure and talent pool necessary to sustain and scale innovations in the long run.

#### (iii) Skilled workforce and continued education

Singapore’s greatest resource is its human capital. A highly skilled workforce adept at driving innovation, advancing research and executing specialized operations in high-tech food manufacturing sectors is central to Singapore’s food security strategy.

#### Skilled workforce

In 2018, Enterprise Singapore (EnterpriseSG), a statutory board under MTI, together with several agencies such as the Economic Development Board, A*STAR, SFA, IPI Singapore and JTC Corporation, came together to launch FoodInnovate [29]. This programme aims to help food companies innovate and grow by providing them with the necessary aid and resources. One of the schemes under FoodInnovate, the Global Ready Talent programme, aims at helping companies to obtain young talents, where subsidies of up to 70% of their monthly stipend are given to companies for hiring student interns from the various institutions across Singapore [30].

Companies seeking experienced professionals to bolster their R&D efforts can use the Innovative & Enterprise Fellowship programme [31]. This programme cultivates a pool of mid-career professionals skilled in technical and commercial development aspects of deep tech commercialization, including product development and market evaluation. Through a combination of formal training in key technological commercialization skills and practical experience, the programme empowers fellows to contribute to R&D efforts and drive innovation within companies [31].

For companies with more urgent R&D needs, the T-Up programme offers a timely solution. Through this initiative, A*STAR scientists and research engineers are seconded to these start-ups and SMEs to assist in product R&D for up to two years. EnterpriseSG provides subsidies to support employee salaries, making this a cost-effective solution for businesses [32].

#### Continued education

To remain competitive in the rapidly evolving food technology landscape, continuous learning is imperative. In 2021, Nanyang Technological University (NTU), in collaboration with the Good Food Institute (GFI), pioneered the first alternative protein university course in the Asia–Pacific (APAC) region. This groundbreaking initiative equips undergraduate students with knowledge on meat alternatives and their processing methods [33]. Similarly, the National University of Singapore (NUS) launched a graduate course in this field [34]. By offering these specialized courses, educational institutions like NTU and NUS are empowering aspiring food scientists to stay abreast of cutting-edge developments in alternative protein technology, ensuring preparedness for industry roles and alignment with current food industry needs.

To further support the existing workforce, SkillsFuture Singapore is a national movement that offers Singaporeans a range of short courses designed to enhance professional skills. One such course, ‘Developing an Innovative Food Business Ecosystem with Global Presence’, focuses on the transformation of the food industry, fostering innovation-driven capabilities and developing a consumer-centric mindset [35]. This course empowers individuals to contribute to and shape the future of the food industry.

Training and development of a skilled workforce is essential for driving innovation and maintaining a competitive edge. Specialized courses, such as those focused on alternative meats and food innovation, provide critical knowledge and skills for professionals in the food industry. On the other hand, programmes that support continuous learning, skill enhancement and career advancement ensure that the workforce remains relevant and proficient to keep pace with the current needs of the ever-changing food industry. By investing in training and development, organizations can foster a culture of innovation, enhance expertise and achieve strategic goals.

### (b) Collaborative partnerships

Strategic public–private partnerships are crucial for accelerating innovation across sectors. By fostering collaboration between government, private companies, research institutions, institutes of higher learning (IHLs) and non-governmental organizations to pool resources and expertise, diverse perspectives and capabilities can be used to drive transformative changes and ensure long-term food security.

#### (i) Public–private partnerships

A notable example of a public–private partnership is the collaboration between a global leader in nutrition, Archer Daniels Midland (ADM) and Nurasa (formerly Asia Sustainable Foods Platform) [36], an innovation platform focused on food technology and sustainable agriculture. In 2022, they established ScaleUp Bio, a joint venture accelerating the commercialization of innovative food products, including alternative proteins. This collaboration uses ADM’s extensive expertise in nutrition and Nurasa’s focus on cutting-edge R&D, reflecting the type of coordinated effort that is essential for achieving Singapore’s food security objectives. Since its inception, ScaleUp Bio has made significant strides, supporting local companies such as Allozymes and Algrow Biosciences in their fermentation processes and attracting international interest from firms like Terra Bioindustries (Canada) and Argento Labs (United Kingdom) [37]. This successful partnership demonstrates how strategic alliances can pool resources and expertise to drive innovation, scale new technologies and quicken market entry.

#### (ii) Partnerships with institutes of higher learning

Partnerships with IHLs play a pivotal role in accelerating innovation and addressing pressing challenges in the food sector. For instance, SingFarms is a new R&D initiative aiming at enhancing the energy and water efficiency of urban farming practices. Funded by the National Research Foundation Singapore, SingFarms, which is driven by NTU and Wageningen University and Research (WUR), sees involvement of NUS, the Republic of Singapore and Temasek Life Science Laboratory (TLL). This initiative is an extension of the long-standing partnership between NTU and WUR in Food Science and Technology education. There is also a similar initiative (the Asian Institute of Modern Agronomy) involving NTU, NUS and TLL on urban farming solutions with an additional focus on nutrition and safety assessment of urban produce. Proteins4Singapore is an inter-institutional collaboration between Technical University of Munich (TUM), NTU, Singapore Institute of Technology (SIT) and A*STAR’s SIFBI. This initiative focuses on developing highly nutritious and functional non-animal proteins through innovative processing techniques and novel reverse food engineering approaches [38]. Likewise, the recent launch of the Bezos Centre for Sustainable Proteins in 2024, led by 23 principal investigators from NUS, NTU, SIT and Eidgenössische Technische Hochschule Zürich [39], underscores the potential of such partnerships to advance alternative protein research. The focus of this centre is on alternative protein research and developing advanced hybrid foods that can rival traditional meat in taste and affordability. These examples illustrate how partnerships with IHLs can facilitate knowledge transfer, talent development and the development of cutting-edge technologies that contribute to sustainable food solutions.

#### (iii) Collaborative spaces

Co-working spaces and collaborative laboratories play a vital role in fostering innovation within the food industry by enabling start-ups and established companies to share resources and expertise. Having access to shared facilities and resources can greatly reduce barriers to entry for start-ups, such as requiring lower capital investments that start-ups need to outlay. These shared environments also foster a culture of innovation by facilitating networking, knowledge exchange and joint research projects among diverse stakeholders in the food industry. Beyond physical infrastructure, many co-working spaces often offer essential support services, including mentorship programmes, business development advice and regulatory guidance. These services help start-ups navigate the complexities of product development and commercialization.

One facility that exemplifies the importance of collaborative spaces is the high-pressure processing (HPP) resource sharing facility [40]. This specialized facility supports innovation in food preservation technologies by providing access to advanced yet costly HPP equipment. HPP is a non-thermal food processing technique that extends shelf life while preserving nutritional quality and flavour [1]. By eliminating the need for significant capital investment in costly equipment, the HPP facility empowers food start-ups and researchers to use this technology and drive innovation in the food industry.

Another great example would be the Food Technology Innovation Centre (FTIC), an initiative by Nurasa, a wholly owned company of Temasek, which serves as a strategic hub for product innovation and commercialization for sustainable foods [41]. It supports research and innovation in food technology, focusing on advanced food processing techniques for scale-up production. The centre currently houses the High Moisture Extrusion joint laboratory, equipped with a pilot-scale set-up capable of producing up to 60 kg of extrudate per hour, as well as the Precision Fermentation joint laboratory, featuring two 100 l food-grade fermentation bioreactors and 15 l per hour spray dryers. To further expand its capabilities, FTIC is actively working on establishing a 10 000 l fermentation capacity and a 50 l per hour spray drying facility in Tuas [29,41]. These state-of-the-art facilities enable researchers and innovators to accelerate product development and bring sustainable food solutions to market.

### (c) Agile regulatory framework and food safety

An agile regulatory framework is essential for supporting food innovation while ensuring safety and compliance. By being flexible, collaborative and risk-based, this approach helps accommodate new technologies and food products. Concurrently, robust food safety practices are critical to protecting public health and maintaining trust in the food supply. Integrating agile regulation with effective food safety measures allows for the safe and efficient development of innovative food solutions.

#### (i) Supportive regulatory environment

An agile and supportive regulatory environment is essential for fostering innovation while ensuring public safety. Singapore’s regulatory bodies, including the SFA, have developed streamlined approval processes using a proactive and risk-based approach to facilitate the rapid development and deployment of new food technologies. This approach enables innovators to bring products to market more quickly while ensuring public safety. By moving away from a one-size-fits-all regulatory framework, Singapore has pioneered innovative solutions. For example, Singapore was the first country to approve the sale of cultured meat, demonstrating its proactive approach to regulating emerging technologies. SFA’s decision was based on a comprehensive risk-based evaluation, including extensive scientific data and safety assessments [42]. The approval process involved close collaboration with Eat Just to ensure safety and regulatory requirements were met. By working closely with stakeholders, regulatory bodies can develop effective and efficient regulatory frameworks that allow innovators to bring products to market more quickly without compromising on safety, ensuring that innovation is not stifled by overly burdensome regulations.

#### (ii) Focus on food safety and traceability

As new food technologies emerge, maintaining high standards of food safety is paramount. Singapore’s commitment to food safety is reinforced by initiatives like the Future Ready Food Safety Hub (FRESH), a collaboration between SFA, A*STAR and NTU [43,44]. Launched in 2021, FRESH aims to build food safety science and R&D capabilities for Singapore's new food innovation ecosystem and contribute to national and global efforts to achieve food security to support the innovation of novel foods [43]. By developing new approach methodologies for novel foods and a New Generation Risk Assessment framework, FRESH enhances the country’s ability to evaluate the safety of new food products efficiently and effectively, bolstering consumer trust by demonstrating a robust commitment to food safety [44].

As a global trading hub, Singapore relies heavily on food imports from over 180 countries. To safeguard public health, the SFA has implemented stringent food safety standards and regulations. Food safety is particularly important in a country like Singapore, where any disruption in the supply chain could have significant public health implications. To address this, the agency uses technology to enhance food safety and traceability across the supply chain, mitigating risks and ensuring the quality of imported food products.

Blockchain technology offers significant potential to enhance food safety and traceability if used effectively. By offering a decentralized and tamper-proof ledger that records every transaction along the food supply chain, from farm to fork [45,46], blockchain enables consumers to trace the origin of their food and verify its safety and quality. Singapore has embraced this technology in 2019, with SFA partnering with ST Engineering and veriTAG, a cloud-based tracking system based on the NULS blockchain platform to ensure food traceability [47]. This system allows stakeholders in the food supply chain, including farmers, processors, distributors and retailers, to record and share information about food products in real time [48]. Consumers can access this information through QR codes on product packaging and verify that the information printed on the labels themselves has not been altered, allowing a level of transparency that empowers consumers to make informed choices and trust in the safety and quality of the food they purchase.

In addition to blockchain, Singapore is also harnessing the potential of internet of things (IoT) devices and artificial intelligence (AI) to further enhance food safety management. IoT devices, such as sensors and smart packaging, can monitor environmental conditions, such as temperature and humidity, throughout the supply chain, enabling real-time tracking and early detection of potential risks like temperature fluctuations or contamination, allowing for immediate action that prevents food spoilage and food safety issues that could result in economic losses [49–51].

AI-driven predictive analytics further strengthens Singapore’s food safety. By analysing historical data and identifying patterns, AI can predict potential food safety issues before they occur, enabling pre-emptive measures to mitigate risks [52]. For instance, AI can forecast the likelihood of a contamination outbreak based on factors such as weather conditions, supply chain disruptions or changes in import/export patterns [52].

A combination of these technological advancements alongside an agile and robust regulatory framework strengthens Singapore’s food safety infrastructure and ensures the availability of safe and high-quality food for its population. This approach not only protects public health but also fosters consumer confidence in innovative food products.

## 3. Areas of innovation

Currently, Singapore produces food locally in three main areas: hen shell eggs, vegetables and seafood. While hen shell has met the 30% self-sufficiency target (31.9%), vegetable and seafood production remain relatively low at 3.2 and 7.3%, respectively [53]. All meat and fruit supplies at present are being imported. Hence, to address these challenges and enhance food security, the Singapore Food Story programme prioritizes alternative protein sources and ramping up aquaculture and vegetable production.

### (a) Alternative proteins

#### (i) Plant-based proteins

Within the alternative protein space, Singapore is well-positioned to lead owing to its cultural, geographical and economic factors. Singapore, being a metropolitan city which began as a tiny fishing village many years ago, has had many migrants of Chinese [54] and Indian [55] descent. These communities have a long-standing history with vegetarian foods stemming from cultural and religious habits. This heritage has fostered a deep understanding of plant-based proteins, such as soy, wheat and pea, as a main protein source. This not only allows for greater knowledge in the use and processing of such proteins but also greatly increases the acceptance and adoption of different plant-based proteins and ensures their translatability across the region owing to shared culinary and dietary preferences.

In addition, Singapore’s strategic location as a global trade nexus, coupled with a stable political environment and robust economic infrastructure, provides a conducive ecosystem for R&D, making it an ideal testing hub for innovative plant-based food products within the Southeast Asia (SEA) and APAC regions. This has attracted global industry leaders to establish research centres and collaborate with local institutions. For instance, Givaudan and Bühler opened a joint Protein Innovation Centre in 2021 with gathered experts from research centres of both companies from across the world. This facility boasts the latest extrusion and processing machines, which could produce up to 40 kg of plant proteins an hour [56]. Other international industry players, such as Bunge, have also established research collaboration agreements with the Food Science and Technology Programme in NTU to develop novel flavour compounds for their plant-based proteins [57]. Furthermore, organizations such as GFI and Bezos Earth Fund have chosen Singapore as their regional headquarters, further solidifying the country’s position as a global hub for alternative protein research.

#### (ii) Insect proteins

The multicultural environment in Singapore, with its strong influence from SEA cultures, has created a receptive audience for insect-based foods. Within SEA, countries such as Thailand and Vietnam are well-accustomed to consuming insects, and the exposure and influence from these neighbouring countries make the acceptance of insect proteins more likely in Singapore. Since July 2024, Singapore has approved 16 different species of insects for human consumption. This regulatory move has paved the way for companies like InsectYumz to import insects from countries like China, Thailand and Vietnam to be processed into various food products. Other companies, such as Altimate Nutrition, have formed joint ventures with international partners like Global Bugs, a Thai insect farm, to develop their own line of food product offerings [58].

#### (iii) Microbial proteins

Within the area of microbial proteins, Singapore uses its established expertise in biotechnology and pharmaceutical manufacturing, which has been likened to fermentation and cellular agriculture processes used in the production of microbial proteins. Companies like TurtleTree Labs are pioneering the use of precision fermentation technologies to produce milk proteins, such as lactoferrin, to make milk. Producing milk in a controlled laboratory environment has been found to be significantly more sustainable in terms of water, land and energy usage compared with conventional farming [59]. Other companies, such as Mycovation and OsomeFood, are exploring the potential of single-cell mycoproteins, cultivated from fungi [60]. On this front, SFA is also the first regulatory body to approve Solein, a type of microbial protein grown with carbon dioxide and electricity, making Singapore a global leader in regulating and adopting novel food technologies [61]. This forward-thinking approach further expresses the commitment and enthusiasm to fostering a thriving alternative protein industry.

#### (iv) Cultured meat

Finally, Singapore’s expertise in biotechnology and biomedical sciences positions it well for the development of cultured meat. The skill sets and processes involved in these fields can be directly transferred to cultured meat production. This is evident in the growing number of cultured meat start-ups in Singapore. Companies such as Umami Bioworks, ImpacFat and Fisheroo have expertise that lies in fish/seafood cell culture, while others such as Meatiply, Ambrosia Sciences and Ants Innovate focus on meat culture [60]. Singapore’s leadership in the approval and commercialization of cultured meat places it at the forefront of world food innovation. The first in the world approval of EatJust’s cultured chicken sets a global precedent for other multinational companies to follow. Meatable, a Dutch cultured meat producer, has also partnered with local contract manufacturer Esco Aster to produce cultivated pork, which could be ready to hit the markets as early as the end of 2024 [62].

Singapore’s efforts are also well aligned with the global trend and development, with NTU actively engaged by the Asian Development Bank, the Food and Agriculture Organization of the United Nations and the World Health Organization for expert advice on the global policy of the above-mentioned novel foods (https://events.development.asia/materials/20220324/keynote-presentation-1; https://www.who.int/publications/i/item/WHO-HEP-NFS-SSA-2024.3.1; https://www.fao.org/food-safety/news/news-details/en/c/1662307/). Singapore’s achievements in this sector are often compared to those of Israel, another innovation-driven nation with limited natural resources. Israel has emerged as a hub for alternative protein start-ups, particularly in the plant-based and cultured meat sectors, with companies like Redefine Meat and Aleph Farms leading the charge [63]. These developments are largely driven by a vibrant private sector supported by government grants and incubators, like Singapore’s ecosystem [64]. However, while Israel’s alternative protein industry is largely driven by private sector initiatives, Singapore’s success can be attributed to a more coordinated approach, involving synergistic public–private partnerships. This ability to integrate government support with industry initiatives has enabled Singapore to accelerate the development and commercialization of alternative proteins, making it a global leader in this space.

On the other hand, the United States (US), home to some of the largest alternative protein companies, including Beyond Meat and Impossible Foods, has significantly popularized plant-based meat substitutes, driven by a strong venture capital ecosystem. However, the US faces challenges with its fragmented regulatory landscape, especially in the cultured meat sector, where state-level discrepancies present challenges to market entry [65,66]. By contrast, Singapore’s streamlined regulatory framework has allowed it to surpass other regions in the approval and commercialization of novel food products. While Singapore could benefit from the entrepreneurial dynamism that characterizes Israel and the US, its cohesive, coordinated approach continues to set a benchmark for innovation in the novel foods space.

### (b) Urban agriculture

In contrast to many of its SEA neighbours, Singapore’s limited land and energy resources place it at a distinct disadvantage. Unlike countries with vast agricultural lands, Singapore must innovate to produce food efficiently within its constrained environment.

#### (i) Vertical farming

Vertical farming reduces the need for large land areas and water resources compared with traditional agriculture. By using hydroponic systems and LED lighting, vertical farms minimize water consumption and maximize energy efficiency, contributing to environmental sustainability. For instance, companies like Sustenir Agriculture have pioneered vertical farming techniques in Singapore that significantly reduce land and water usage. Sustenir Agriculture focuses on growing non-native crops, such as kale and strawberries, which are typically imported [67]. This aids in reducing Singapore’s reliance on food imports and contributes to the country’s food security.

However, energy costs in Singapore are not subsidized, complicating efforts to develop and maintain energy-intensive agricultural technologies such as vertical farms and controlled environment agriculture. This stands in stark contrast to neighbouring countries, where lower energy costs and vast areas of land can make large-scale farming more economically viable.

Land and resource scarcities have led to a focus on maximizing productivity per unit area, and urban farming techniques like vertical farming are emerging as a promising solution to some of Singapore’s land constraints. Singapore’s approach to urban farming shares common ground with The Netherlands and Japan, both of which have embraced advanced technologies such as vertical farming to address food security amidst land constraints. The Netherlands, a global leader in agricultural innovation, has developed some of the world’s most advanced vertical farming technologies, supported by robust governmental backing in agricultural research [68,69]. Despite its small land area, The Netherlands ranks among the top agricultural exporters globally, producing approximately €65 billion in agricultural produce annually, constituting 17.5% of its total exports [70]. Similarly, Japan, with its dense urban population and limited arable land, has focused on highly efficient urban farms that use LED lighting and automation [71]. These farms are pivotal to Japan’s food security, enabling the production of a diverse array of crops, from leafy greens to fruits, within urban settings [72].

#### (ii) Farming within urban areas

While Singapore’s vertical farming efforts are adapted from and share many similarities with those in The Netherlands and Japan, particularly in the use of technology to overcome land limitations, Singapore distinguishes itself through its strategic integration of vertical farms into the urban landscape, reflecting a commitment to building a resilient and sustainable food system. For example, Frux Earth, a Singaporean company that specializes in urban farming and aquaponics, started a 1110 sq m rooftop aquaponics farm near the city centre. Following its success, the company expanded to having more than 5575 sq m of rooftop aquaponics farms all across the country [73]. This unique strategy not only addresses Singapore’s land scarcity issues but also positions it as a pioneer in urban agriculture within densely populated environments [74].

#### (iii) Challenges and opportunities

However, the high operational and energy costs, coupled with the need for specialized knowledge and large capital outlay, present significant barriers to widespread adoption and scalability. Given the high reliance on robotics, LED lighting and complex irrigation and nutrient systems, urban farming companies state energy cost as the largest contributor to their operational expenses. Coupled with high rental and labour costs, it can be challenging to sustain the business, resulting in the exit of some companies, such as I.F.F.I [75]. Another challenge is the consumer acceptance of these novel foods, which is not limited to Singapore and needs proactive education and integrated approaches to create a new food value chain for the sustainability of these urban solutions.

Despite the challenges, some companies have successfully adapted and thrived in the urban agriculture landscape. Artisan Greens, for instance, had implemented innovative strategies and systems to reduce energy consumption and has seen tremendous growth, achieving profitability since early 2022 and looking to diversify their produce offerings [76]. Growy, the Dutch agritech company that started the first commercially viable large-scale vertical farm in The Netherlands, recognized the potential of Singapore’s urban farming market and established a large-scale vertical farm in 2023. Since then, they have achieved notable success and are expanding their operations to include a second facility. Laura Van de Kreeke, second-generation farmer at Growy, reiterated the importance of efficient energy management for long-term commercial viability [77]. Other international companies are also recognizing the potential of Singapore’s urban agriculture market. Future Food Foundry, a United Arab Emirates (UAE)-based company, invested USD 20 million in 2024 to acquire and merge two local farms, Sustenir and NextGen Farms. The company aims to create an international network of high-tech farms focused on producing superfoods like kale, using its experience in addressing similar food security challenges in the UAE [78].

### (c) Aquaculture and sustainable seafood

Aquaculture is a significant focus of Singapore’s sustainable food production efforts. Given the limited land availability and being an island nation, Singapore has driven its focus on sustainable aquaculture. As part of the Singapore Aquaculture Plan, the AquaPolis research programme was introduced in 2022 to support and promote R&D in the aquaculture sector [25].

#### (i) AquaPolis

The AquaPolis programme brings together research institutes, IHLs and industry players to address current challenges in aquaculture, such as improving fish health, growth rates and nutrient content in fishes, i.e. improving omega-3 content in fishes. The programme encompasses three key areas across the country at the East and West Johor Straits, as well as the Southern Waters. Within this programme, SFA’s Marine Aquaculture Centre serves as a shared facility that provides resources like replicated tank systems, eggs, larvae, rotifers and microalgae for research. Researchers can leapfrog from shared husbandry-related expertise, facilities and resources to conduct cutting-edge aquaculture research and translate their findings into practical applications.

#### (ii) Technology in aquaculture

To enhance farming practices and productivity, Singapore’s aquaculture industry is adopting innovative technologies. Offshore aquaculture, a key focus area, allows for the cultivation of fish and seafood in open sea environments. Sustainable aquaculture practices, supported by innovations in high-density offshore systems, are aligned with global best practices, contributing to the long-term viability of Singapore’s food system.

To achieve this goal, the Aquaculture Centre of Excellence (ACE), a public–private partnership, leads the development of innovative aquaculture systems. ACE has developed and produced the world’s first purpose-built, closed-containment floating fish farm, Eco-Ark. Equipped with advanced technologies like seawater filtration, ozone sterilization, vortex systems to mimic ocean currents and recirculating aquaculture systems (RAS), a technology enabling the recycling of water and nutrients to reduce environmental impact, this innovative facility minimizes environmental impact while maximizing productivity. By cultivating fish from eggs in a closed-containment environment, ACE prevents infections and contamination, lowering mortality rates and eliminating the need for antibiotics. Additionally, the facility’s 100% solar-powered and IoT-enabled monitoring systems contribute to its sustainability. ACE is also exploring integrated multi-trophic aquaculture, a method that combines the farming of fish with other species, such as shellfish and seaweed, to create a balanced ecosystem. This approach not only increases productivity but also enhances the sustainability of aquaculture by reducing waste and improving water quality [26,79].

#### (iii) Singapore’s unique challenges

When considering global leaders in sustainable aquaculture, Norway and New Zealand are frequently highlighted as pioneers, setting benchmarks for responsible seafood production. Norway, a world leader in farmed salmon, has pioneered innovative technologies to minimize environmental impact and enhance fish welfare. Closed-containment systems and offshore fish farms, for example, offer effective solutions for reducing the ecological footprint [80]. Similarly, New Zealand, renowned for its pristine marine environment, has integrated sustainable aquaculture into its food security strategy. The country’s rigorous environmental regulations and best practices ensure that its aquaculture industry operates in harmony with the natural ecosystem. By incorporating indigenous knowledge and fostering community engagement, New Zealand adopts a holistic approach in marine resource management [81].

Together, Norway and New Zealand serve as exemplary models for sustainable aquaculture, demonstrating how technological advancements and environmentally responsible practices can coexist to produce high-quality seafood while safeguarding the environment. Their vast land areas, abundant natural resources and favourable climates enable large-scale, sustainable aquaculture operations for larger species like salmon and cod. By contrast, Singapore faces significant challenges. Aside from limited land and water resources, high energy costs and tropical climate constraints necessitate innovative approaches to aquaculture. By drawing inspiration from the successes of Norway and New Zealand, while adapting to its unique context, Singapore can pioneer sustainable urban aquaculture. Conscientious efforts must be made to incorporate new research and technology to overcome these issues, as has been demonstrated by ACE. Hence, the Singapore Aquaculture Plan serves to provide this concerted push to help companies in this endeavour. This will not only contribute to Singapore’s food security but also provide a model for other densely populated regions facing similar constraints in urban aquaculture.

## 4. Conclusion

Singapore’s food innovation landscape is a dynamic and evolving ecosystem that reflects the country’s steadfast commitment to food security, sustainability and public health. Through strategic investments in alternative proteins, urban farming, food safety technologies and sustainability initiatives, Singapore has positioned itself as a global leader in food innovation.

The country’s success is underpinned by a proactive and supportive government, a robust regulatory framework and a collaborative approach that brings together public and private sectors. These elements have enabled Singapore to overcome its resource limitations and build a resilient food system that can serve as a model for other countries facing similar challenges.

However, the journey towards food security and sustainability is an ongoing one. As global challenges such as climate change, population growth, geopolitical tensions and resource scarcity continue to evolve, Singapore’s ability to pivot quickly, test and refine new technologies and move swiftly from concept to implementation is a distinct advantage. As a small yet agile nation, Singapore not only fortifies its own food security but also establishes itself as a test bed for innovations that could benefit other cities and countries with similar constraints.

Looking forwards, continuous investment in R&D, coupled with the adoption of cutting-edge technologies, will be essential in maintaining Singapore’s leadership in food innovation. In doing so, Singapore will be able to continue enhancing the resilience and sustainability of its food system in an increasingly uncertain global landscape.

## Data Availability

This article has no additional data.
